# Single session of intermittent theta burst stimulation alters brain activity of patients in vegetative state

**DOI:** 10.18632/aging.205746

**Published:** 2024-04-18

**Authors:** Ying Huang, Xiaoyu Xia, Xiangqiang Meng, Yang Bai, Zhen Feng

**Affiliations:** 1 The Affiliated Rehabilitation Hospital, Jiangxi Medical College, Nanchang University, Nanchang 330003, Jiangxi, China; 2 Rehabilitation Medicine Clinical Research Center of Jiangxi Province, Nanchang 330003, Jiangxi, China; 3 Department of Neurosurgery, The Seventh Medical Center of PLA General Hospital, Beijing 100700, China

**Keywords:** intermittent theta burst stimulation, vegetative state, permutation entropy, weighted phase lag index

## Abstract

Background: Non-invasive brain stimulation is considered as a promising technology for treating patients with disorders of consciousness (DOC). Various approaches and protocols have been proposed; however, few of them have shown potential effects on patients with vegetative state (VS). This study aimed to explore the neuro-modulation effects of intermittent theta burst stimulation (iTBS) on the brains of patients with VS and to provide a pilot investigation into its possible role in treating such patients.

Methods: We conducted a sham-controlled crossover study, a real and a sham session of iTBS were delivered over the left dorsolateral prefrontal cortex of such patients. A measurement of an electroencephalography (EEG) and a behavioral assessment of the Coma Recovery Scale-Revised (CRS-R) were applied to evaluate the modulation effects of iTBS before and after stimulation.

Results: No meaningful changes of CRS-R were found. The iTBS altered the spectrum, complexity and functional connectivity of the patients. The real stimulation induced a trend of decreasing of delta power at T1 and T2 in the frontal region, significant increasing of permutation entropy at the T2 in the left frontal region. In addition, brain functional connectivity, particularly inter-hemispheric connectivity, was strengthened between the electrodes of the frontal region. The sham stimulation, however, did not induce any significant changes of the brain activity.

Conclusions: One session of iTBS significantly altered the oscillation power, complexity and functional connectivity of brain activity of VS patients. It may be a valuable tool on modulating the brain activities of patients with VS.

## INTRODUCTION

After severe brain injury and moving out of a coma, some patients may evolve into a state of disorders of consciousness (DOC) where they display minimal to no responsiveness to external stimuli, often coupled with deficits in motor and sensory abilities [1]. Among them, patients with vegetative state (VS) preserved behavioral arousal, but lacked awareness [2]. Patients with minimally conscious state (MCS) have fluctuating yet reproducible remnants of non-reflex behaviors [3]. The large population of DOC patients has brought tremendous challenges to the clinics, as they are communication-disabled and are dependent on others for care. With regards to therapeutic options, only a few studies have investigated the treatment of patients with DOC. Pharmacological interventions, including amantadine, intrathecal baclofen, and zolpidem, have been utilized to enhance consciousness and functional recovery in patients with disorders of consciousness. Among these, only amantadine has been supported by class II evidence. In addition to pharmacotherapy, non-pharmacological approaches such as neuromodulation techniques have been explored to improve consciousness and functional outcomes in these patients [4]. The lack of evidence-based effective pharmacological treatment options for DOCs highlights the need of alternative neuromodulator treatments [5].

Studies have investigated the possibility of using neuromodulator technologies in the treatment of DOC patients [5–7]. These technologies could be divided into two categories based on invasiveness, i.e., requirement of surgery: invasive brain stimulation and non-invasive brain stimulation (NIBS). Since it lacks surgical risks, is less costly and has fewer ethical limitations, NIBS increasingly attracts attentions from neuroscientists and clinicians. The most promising technologies includes the transcranial direct current stimulation (tDCS) [8, 9] and repetitive transcranial magnetic stimulation (rTMS) [10, 11].

A number of patients with MCS have benefited from the tDCS and rTMS treatment. Such technologies, however, always exhibit weakness with the VS patients [4]. A long-term crossover study verified a significant treatment effect of tDCS on 43% of patients with MCS [12]. But, for VS patients, no treatment effects were found at the group level. The electrophysiological measurement also showed that tDCS could significantly modulate the neural activities of patients with MCS, but not patients with VS [13, 14]. This is still the case in rTMS treatment. Our previous study found that most patients with MCS could get notable improvement of consciousness after several sessions of rTMS. But only a few patients with VS showed a marked response to the treatment [15]. Therefore, developing new approaches and protocols is a crucial move to seek a treatment strategy for patients with VS.

Theta burst stimulation (TBS) is a modified form of rTMS. It is an effective way to alter human cortical excitability, and it takes less time to apply protocols, making it more acceptable for participants than rTMS [16, 17]. TBS application involves the delivery of 600 pulses within a brief duration of 40 seconds to three minutes, in stark contrast to the extended 18 to 25 minutes typically needed for rTMS protocols. Nevertheless, TBS yields antidepressant outcomes on par with those of rTMS [18, 19]. TBS includes two different paradigms: continuous TBS (cTBS) and intermittent TBS (iTBS), involving pulses applied in bursts of three at 50Hz with an inter-burst interval at 5Hz. cTBS reduces cortical excitability while iTBS increases it. Over the last 10 years, TBS has been increasingly verified as a powerful neuromodulator with therapeutic intent in patients with various types of neurogenic and mental disorders [20–22]. A case report found that a patient with a right frontal lobe glioblastoma had decreased consciousness after surgery and no improvement with other treatments. After receiving five rounds of iTBS treatment, their level of consciousness improved [23]. Recently, a pilot research reported that one patient with VS obtained conscious recovery during an iTBS protocol [24]. This suggested that iTBS may be a possible promising approach for the treatment of patients with VS. But, no one further pursued its underlying effects on such patients. Therefore, the present study combines electroencephalography (EEG) and behavioral scales to explore the responses of patients with VS in iTBS. It would improve our understanding about the electrophysiological mechanism of iTBS on the neural activities of patients with VS. And, it will provide an explorative foundation for following long-term clinical trials to seek treatment strategies based on iTBS for such patients.

## MATERIALS AND METHODS

### Participants

We enrolled medically stable patients with prolonged VS (after brain injury, the patient remains in a vegetative state lasting for at least 28 days) in this study. The exclusion criteria were: contraindications for MRI scans and iTBS applications (e.g. pacemakers, aneurysm clips, nerve stimulators, or brain/subdural electrodes), severe cerebral atrophy or injury in the left frontal area upon MRI scans, a history of epilepsy or EEG epileptiform activity. Finally, 18 patients (8 females and 10 males, average age: 42.38 ± 13.57, average time since injury: 5.75 ± 2.38 months) completed the experiment. Their clinical and demographic characteristics of the patients are shown in Table 1. All the patients were assessed by one week’s repeated Coma Recovery Scale-Revised (CRS-R) process [25]. The CRS-R were conducted by trained neurologists at least three times during the afternoon. All the patients received routine medication and rehabilitation courses but did not obtain consciousness improvement for at least two weeks. They had not taken any drugs such as zolpidem, modafinil, midazolam or baclofen, and were free of any acute medical complications (e.g. acute pneumonia) for at least two weeks. Written informed consent to participate in the study was obtained from the patients’ caregivers. This study was approved by the ethics committee of the Seventh Medical Center of PLA General Hospital. Clinical register: ChiCTR1800017623.

**Table 1:** Demography information of the patients enrolled in the study.

**Index**	**Gender/age (years old)**	**Etiology**	**Interval since insult (month)**	**CRS-R (Auditory/visual/motor/oromotor/communication/arousal)**	**Total CRS-R score**
1	M/43	Anoxia	7	0/0/2/0/0/2	4
2	M/57	Subarachnoid hemorrhage	4	1/0/2/1/0/2	6
3	F/62	Cerebrovascular accident	4.5	0/0/2/1/0/2	5
4	F/38	Anoxia	8.5	1/0/2/1/0/2	6
5	M/39	Subarachnoid hemorrhage	4	1/0/2/1/0/2	6
6	M/53	Cerebrovascular accident	3	0/0/1/0/0/1	2
7	F/42	Anoxia	3.5	1/1/2/1/0/1	6
8	F/18	Cerebrovascular accident	3	1/0/2/0/0/1	4
9	F/33	Trauma brain injury	10	0/0/1/1/0/2	4
10	M/55	Trauma brain injury	5	1/1/2/1/0/2	7
11	M/41	Trauma brain injury	9	0/0/2/1/0/2	5
12	M/20	Anoxia	6.5	1/0/2/1/0/2	6
13	M/26	Anoxia	5.5	1/0/2/1/0/2	6
14	F/43	Subarachnoid hemorrhage	4	0/0/2/1/0/2	5
15	F/53	Subarachnoid hemorrhage	2.5	1/1/2/1/0/2	7
16	M/66	Trauma brain injury	6.5	1/0/2/1/0/1	5
17	M/32	Subarachnoid hemorrhage	8	0/0/2/1/0/1	4
18	F/42	Trauma brain injury	9	0/1/2/1/0/2	6

Abbreviation: M, male; F, female; CRS-R, Coma Recovery Scale-Revised.

### Experimental protocol

**Figure 1:**
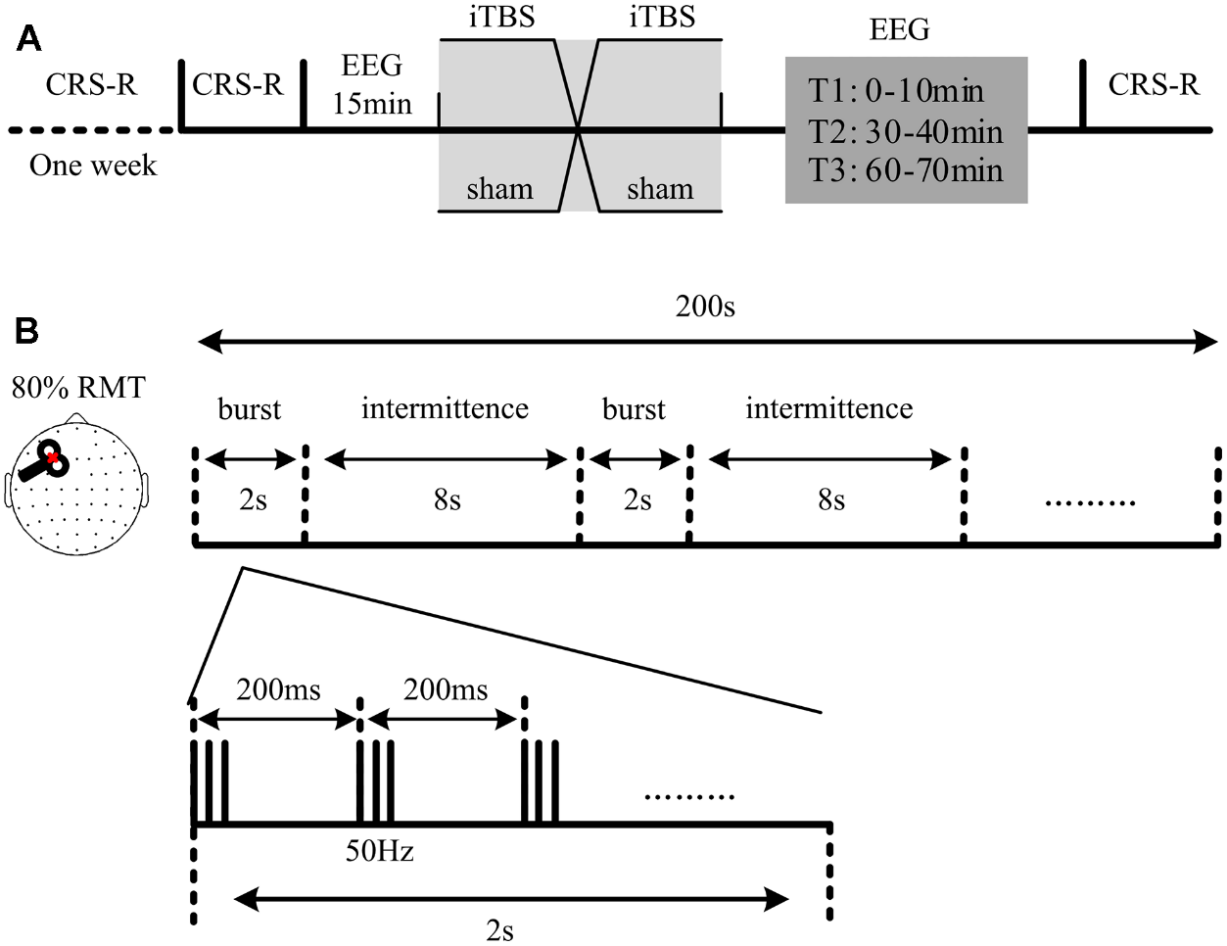
**Protocol of the experiment.** (**A**) Structure of the sham-controlled, cross-over study. Each patient received two randomized sessions of stimulation (a real and a sham session). The real and sham sessions had at least a three-day washout period in between. The Coma Recovery Scale-Revised (CRS-R) assessment and fifteen minutes of electroencephalography (EEG) recording were conducted at the beginning of each session. 70 minutes of EEG recording, followed by another CRS-R assessment, was recorded immediately after each stimulation. T1, T2 and T3 refer to the EEG recording at 0-10 min, 30-40 min and 60-70 min, respectively. (**B**) Intermittent theta burst stimulation (iTBS) setup used in the study. Each stimulation included 600 stimuli: 20 trains of 50Hz bursts (3 pulses) repeated at 5Hz.

A randomized double-blind, crossover experimental was set up (Figure 1A). Each patient received both a real and sham iTBS in a randomized order. A computer-generated randomization sequence was used to assign, the first session as either a real or sham stimulation. For each patient, the experimenter received two blinded codes from a third person, one for the real stimulation and one for the sham stimulation. Before each stimulation, patients received CRS-R and fifteen minutes of resting-state EEG recording. In order to capture the modulation effects of iTBS on patients’ brain activities, 70 minutes of resting-state EEG followed by CRS-R was conducted immediately after the stimulation. The real and sham stimulations had at least a three-day washout period in between. To evaluate the long-term functional outcomes, we followed up with the Glasgow Outcome Scale Extension (GOS-E) scores at 6 months post-experiment via a phone call.

### Stimulation

As shown in Figure 1B, iTBS with an intensity of 80% resting motor threshold (RMT) was conducted at the left dorsolateral prefrontal cortex (DLPFC) of patients. The iTBS included 20 trains of 50Hz bursts (3 pulses) repeated at 5Hz. They were applied at every 10 seconds (total 600 stimuli). The magnetic stimulation was administered in accordance with safety guidelines [26]. TMS pulses were delivered using a Magstim *R*^2^ stimulator with a 70 mm figure-of-eight coil (Magstim Company Limited, Whitland, UK). Stimulation intensities varied across this experiment and were determined relative to the RMT, defined as the lowest TMS intensity that can be evoked in at least five out of 10 trials of an electromyogram with an amplitude >50*μV* peak-to-peak in the relaxed first dorsal interosseous muscle of the right hand. For the real iTBS session, the coil was positioned tangentially (perpendicularly in sham session) to the scalp pointing in an anteromedial direction, 45° from the midsagittal axis of patient’s head.

### EEG recordings and pre-processing

In this study, the EEG recordings were acquired from patients by 62 channels (BrainAmp 64 MRplus, BrainProducts) with positions of the international 10-20 system. The equipment used sintered Ag/AgCl-pin electrodes. We set a band-pass filtered at DC to 1000Hz in the recorder. The EEG signals were digitized at a sampling rate of 2.5 kHz. During the recording, the skin/electrode impedance was maintained below 5kΩ. We monitored patients for possible EEG signs of drowsiness and sleep onset (an increase of tonic theta rhythms, sleep spindles). An arousal procedure of CRS-R would be performed in the patients who showed above EEG signs.

An off-line analysis was performed with the EEGLAB 12.0.2.5b, running in a MATLAB environment (Version 2013b, MathWorks Inc., Natick, USA). An independent component analysis (ICA) function was used to identify and remove the artefact’s relevant components such as eye movement and muscle activities [27]. The EEG data from two patients were excluded because of serious artefacts (over one of three ICA components were identified as artefacts). The EEG data were down-sampled to 500Hz, bandpass filtered (1-45Hz) and average referenced. Then, the EEG data were divided into epochs of 10 seconds. Artefact-free epochs were preserved and recorded for EEG analysis.

### EEG analysis

Spectrum power, nonlinear neural dynamics of permutation entropy (PE) and the functional connectivity of the weighted phase lag index (wPLI) were measured for the EEG at different time points (baseline, T1, T2 and T3). For patients, the PE and wPLI at each time point were calculated by averaging the indexes within the epochs.

### Permutation entropy

PE is a quantitative complexity measure that explores the local order structure of a dynamical time series. It transforms given time series into series of ordinal patterns, each describing the order relation between the present and a fixed number of equidistant past values at a given time [28].

The scalar time series are given as {*x*(*i*) : 1 ≤ *i* ≤ *N*}. Firstly, the reconstruction time series is given as the following;

$$X_{i}=\{x(i),x(i+\tau ),...,\;x(i+(m-1)\tau )\},\;i=1,2,...\;N-(m-1)\tau$$ (1)

where *τ* is time delay and *m* is the embedding dimension.

Then, rearrange *X_i_* in an increasing order:

$$\left\{x(i+(j_{1}-1)\tau )\leq x(i+(j_{2}-1)\tau )]\leq \ldots \leq x(i+(j_{m}-1)\tau )\right\}$$ (2)

There are *m*! permutations for *m* dimensions. Each vector *X_i_* can be mapped to one of the *m*! permutations.

Next the probability of the *jth* permutation occurring *p_j_* can be defined as:

$$p_{j}=\frac{n_{j}}{\sum_{j=1}^{m!}n_{j}}$$ (3)

where *n_j_* is the number of times the *jth* permutation occurs.

The permutation entropy of the time series {*x*(*i*) : 1 ≤ *i* ≤ *N*} is defined by

$$H_{x}(m)=-\sum_{j=1}^{m!}p_{j}\ln p_{j}$$ (4)

when the time series is random, the *H_x_* (*m*) approaches its maximum value of ln(*m*!); when the time series is regular, the *H_x_* (*m*) approaches to zero.

Finally, normalizing *H_x_* (*m*) by diving ln(*m*!):

$$PE=\frac{H_{x}(m)}{\ln(m!)}$$ (5)

### Weighted phase lag index

The wPLI is a conservative measure of phase synchronisation between electrodes [29]. It enables the analysis of the properties of phase synchronization without the deleterious impact of volume conduction. Here, we assumed that wPLI could be used to quantify the modulation effects of iTBS on neural dynamics reflected in the phase oscillatory activity of the scalp EEGs. The wPLI were computed for each EEG channel across the other channels based on the following equation:

$$\begin{array}{lll} {\text{wPLI}}_{i,j} & = & \frac{\left|E\left\{ℑ\{X_{i}X_{j}^{*}\}\right\}\right|}{E\left\{\left|ℑ\{X_{i}X_{j}^{*}\}\right|\right\}}\\ & = & \frac{\left|E\{|ℑ\{X_{i}X_{j}^{*}\}|sgn[ℑ\{X_{i}X_{j}^{*}\}]\}\right|}{E\{|ℑ\{X_{i}X_{j}^{*}\}|\}} \end{array}$$ (6)

where i and j are channel indices, *X*_i_ is the time-frequency spectrum of channel *i*, $X_{j}^{*}$is the complex conjugate of *X_j_*, $ℑ\{X_{i}X_{j}^{*}\}$indicates an imaginary section of cross frequency spectra $\left\{X_{i}X_{j}^{*}\right\}$, *E*{.} is the expected value operator and *sgn*{.} is the sign function operator.

### Statistic

A statistical analysis was performed using SPSS (version 17.0). The effect of the stimulation on consciousness state was analyzed based on the modification of the CRS-R total score, using the paired t-test. The modification of the brain activity was assessed by comparing EEG indexes between different time points in each stimulation session. The differences were checked by one-way repeated ANOVA. Post-hoc paired t-tests were used in the comparison of different time points: T1 vs. baseline, T2 vs. baseline, and T3 vs. baseline. Bonferroni correction was performed after multiple comparisons. The comparisons of PE values and functional connectivity at sensor level were conducted by the paired t-tests with FDR correction.

## RESULTS

### CRS-R

No significant difference (p > 0.05, paired t-test) of total CRS-R scores between before and after stimulation (Table 2), either in the real or sham sessions. Some patients showed fluctuation of total CRS-R scores, but none of the patients gained an improvement of consciousness state. Patient 6 and patient 16 showed a change in the arousal sub-scale, showing stable sign of slight spontaneous opening eyes without external stimulation (2 scores in arousal sub-scale) after real stimulation. None of the other patients displayed any valuable changes of CRS-R scores exceeding those in the previous one week’s diagnosis.

**Table 2:** Coma recovery scale-revised scores of the patients assessed at before and after stimulation and the Glasgow outcome scale extension scores at 6 months post-experiment.

**Patients**	**(Auditory/visual/motor/oromotor/communication/arousal)-total score**				**Glasgow outcome scale extension scores**
	**Before real**	**After real**	**Before sham**	**After sham**	
1	(001002)-3	(002002)-4	(002002)-4	(002002)-4	1
2	(102102)-6	(102102)-6	(102102)-6	(101102)-6	1
3	(002102)-5	(002102)-5	(002102)-5	(002102)-5	1
4	(102102)-6	(102102)-6	(102102)-6	(102102)-6	1
5	(002102)-5	(002102)-5	(102102)-6	(002102)-5	1
6	(001001)-2	(001002)-3	(001001)-2	(001001)-2	2
7	(102101)-5	(112101)-6	(112101)-6	(112101)-6	2
8	(102001)-4	(102001)-4	(102001)-4	(102001)-4	1
9	(001102)-4	(001102)-4	(001102)-4	(001102)-4	1
10	(102102)-6	(112102)-7	(102102)-6	(102102)-6	2
11	(002102)-5	(002102)-5	(002102)-5	(002102)-5	1
12	(102102)-6	(102102)-6	(102102)-6	(102102)-6	1
13	(002102)-5	(002102)-5	(002102)-5	(102102)-6	2
14	(002102)-5	(002102)-5	(002102)-5	(002102)-5	1
15	(112102)-7	(112102)-7	(112102)-7	(112102)-7	1
16	(102101)-5	(102102)-6	(102101)-5	(102101)-5	3
17	(002101)-4	(002101)-4	(002101)-4	(002101)-4	1
18	(012102)-6	(012102)-6	(112102)-7	(012102)-6	1

### Spectrum

**Figure 2:**
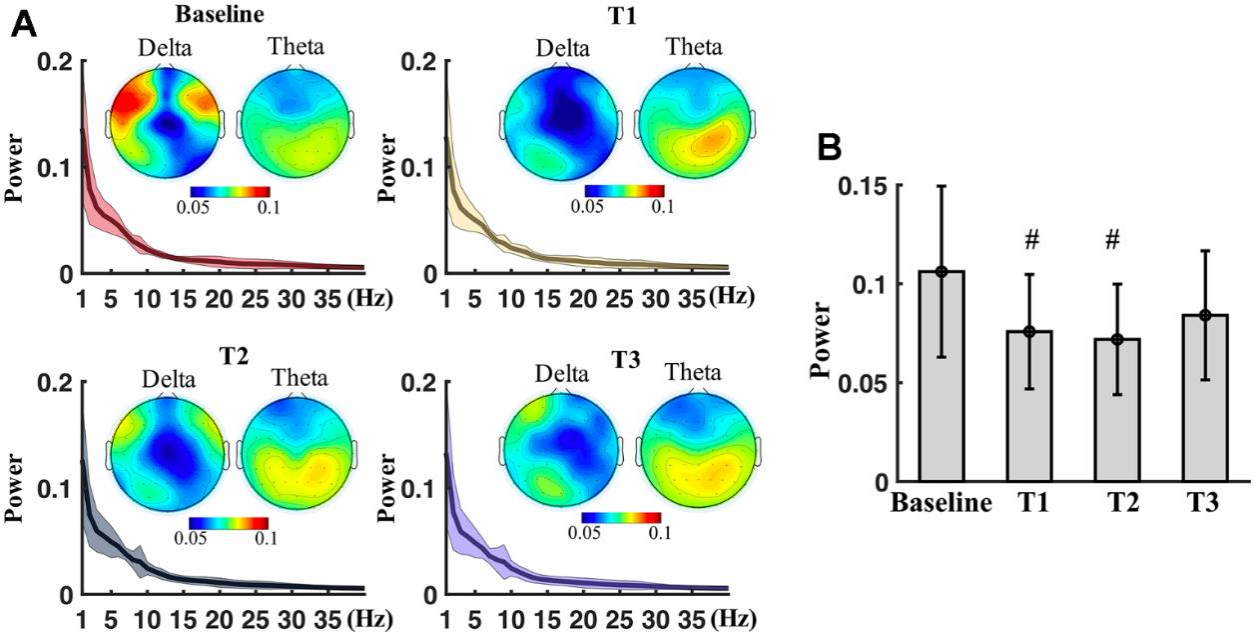
**Spectral power measured at different time points.** (**A**) Spectrum power (mean ± SD) averaged from all electrodes at baseline, T1, T2 and T3. Topography shows spatial distribution of delta and theta power at the time points. (**B**) Boxplots of the average power of delta of frontal region at baseline, T1, T2 and T3. # means significance before multiple comparison correction but non-significance after correction.

There was no significant difference of global average spectrum between T1, T2, T3 and baseline in the real stimulation. Topography indicated a decreasing of delta power and an increasing of theta power (Figure 2A). There was no significance of the frontal average delta power among the different time points (F_3,71_ = 3.45, p>0.05). No significance in the pairwise comparisons of the frontal average delta power. However, the frontal average delta power of T1 (p = 0.03) and T2 (p = 0.04) were significantly lower than that of baseline before multiple comparison correction (Figure 2B). Sham stimulation did not induce any significant changes of spectrum power of EEG.

### Permutation entropy

**Figure 3:**
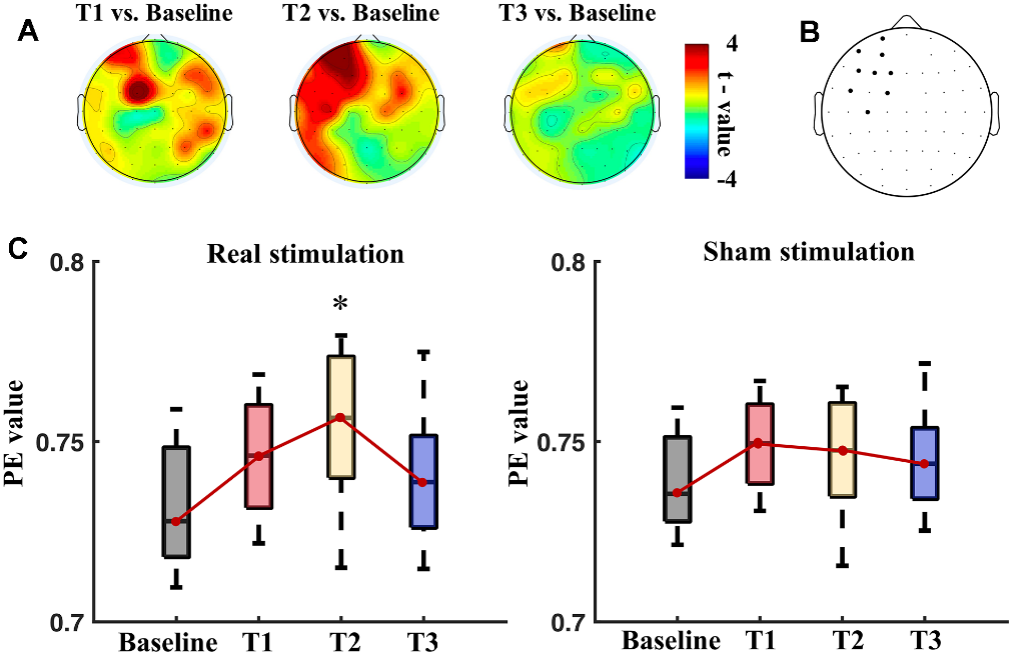
**Permutation entropy (PE) values measured at different time points: baseline, T1, T2 and T3.** (**A**) Topography of t values of paired t-tests in comparisons of PE values of T1 vs. baseline, T2 vs. baseline, and T3 vs. baseline. (**B**) Black dots show electrodes that had significant difference (p<0.05, paired t-tests with FDR correction) of PE values between T2 and the baseline. (**C**) Boxplots of the PE values (left panel: real session, right panel: sham session) averaged from the left frontal region at the baseline, T1, T2 and T3 in the real and sham sessions. * means significance in the one-way repeated ANOVA with post-hoc t-tests after Bonferroni correction.

PE values were measured at the baseline and the post-stimulation time points. Then, PE values at the T1, T2 and T3 were compared with that of the baseline for the electrodes. The comparisons indicated that electrodes of bilateral frontal and right parietal regions showed significantly different PE values (p<0.05, paired t-tests with FDR correction) between T1 and baseline (Figure 3A). PE values of electrodes at left hemisphere were significantly different between T2 and baseline. None of electrodes showed marked different PE values at T3 in comparison with baseline. Electrodes (FP1, AF3, AF7, F1, F3, F5, FC1, FC5 and C3) have significantly higher PE values (p<0.05, paired t-tests with FDR correction) between the T2 and the baseline (Figure 3B).

Figure 3C shows the boxplots of average PE values of the left frontal region (including FP1, AF3, AF7, F1, F3, F5, FC1, FC5 and C3) of the patients at different time points. There was significant difference between time points in the real stimulation (F_3,71_ = 5.12, p = 0.008), but not in the sham stimulation (F_3,71_ = 1.69, p > 0.05). Post-hoc t-tests showed a significant increase of the PE values at T2 than the baseline (p=0.003, paired t-tests with Bonferroni correction) after the real stimulation. The sham stimulation did not significantly alter the average PE values of the left frontal region.

### Weighted phase lag index

**Figure 4:**
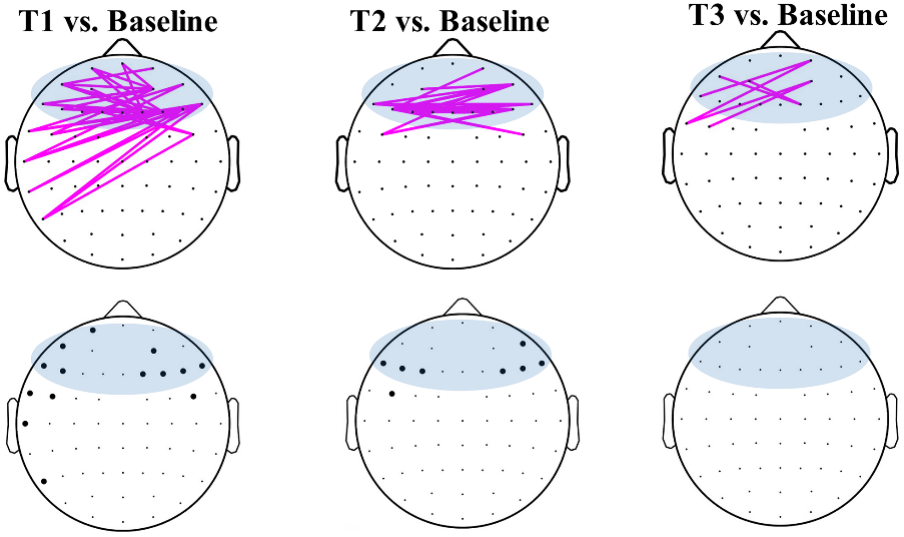
**Weighted phase lag index (wPLI) of pairwise electrodes in the baseline and post-stimulation of the real stimulation.** Pink lines show significantly (p<0.05, FDR correction) strengthened connectivity of T1 (left), T2 (middle) and T3 (right) in comparison with baseline. Blue areas show electrodes defined in the frontal region. The black dots at lower panel represented the electrodes that showed significant (p<0.05, FDR correction) difference of connectivity degree in time comparisons.

The functional connectivity was indexed by wPLI and measured at the baseline and post-stimulation time points for the pairwise electrodes. Figure 4 shows the significantly strengthened connectivity by the real stimulation (p<0.05, paired t-tests with FDR correction). The significantly strengthened connectivity were mostly relevant with electrodes at frontal region. Consistently, the electrodes, which showed significant increasing of connectivity degree, were mainly located at bilateral frontal regions (lower panel of Figure 4) at T1 and T2.

**Figure 5:**
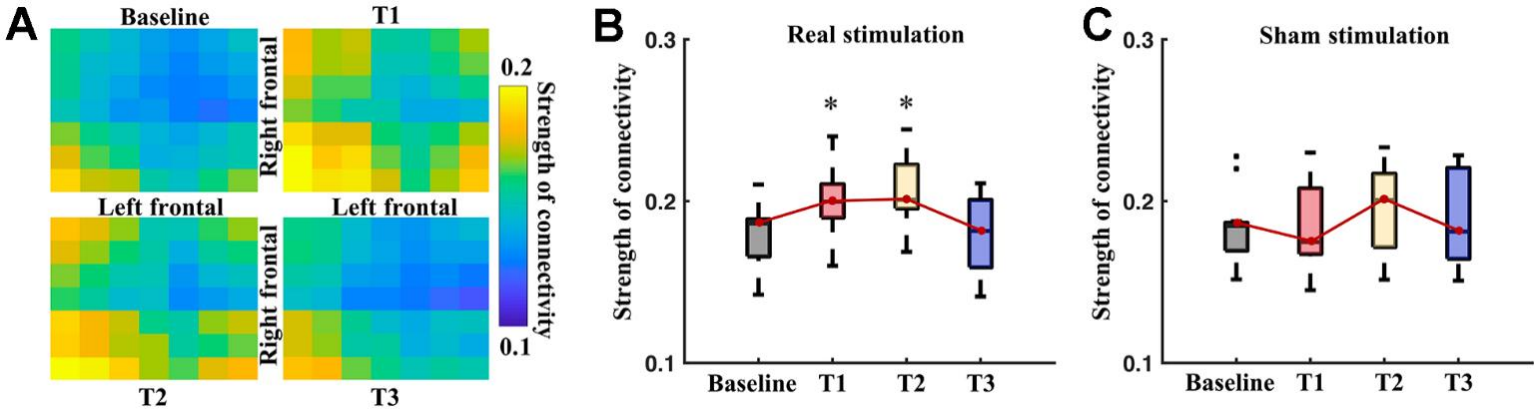
**Inter-hemisphere connectivity of the frontal regions.** (**A**) Pairwise connectivity of inter-hemispheric frontal regions at different time points. Boxplots show average strength of inter-hemisphere connectivity of the frontal region at different time points of real (**B**) and sham (**C**) stimulation. * means significance in the comparison between T1, T2, T3 and baseline, using one-way repeated ANOVA with post-hoc t-tests after Bonferroni correction.

Inter-hemispheric connectivity of frontal regions showed marked increasing at T1 and T2 in comparison with baseline (Figure 5A) after the real stimulation. The average strength of frontal inter-hemispheric connectivity showed significant difference in the time points of the real stimulation (F_3,71_ = 17.33, p < 0.001), but not in the sham stimulation (F_3,71_ = 0.89, p > 0.05). Post-hoc t-tests showed a significant increase at T1 (p=0.03, paired t-tests with Bonferroni correction) and T2 (p=0.008) after real stimulation than that at the baseline (Figure 5B). There was no significant difference between T3 and baseline in the real stimulation. The sham stimulation did not induce any significant change of the inter-hemispheric connectivity at the T1, T2 and T3 between that of the baseline (Figure 5C).

### GOS-E

The functional outcomes of vegetative state patients at 6 months post-experience, as reflected in the GOS-E scores (Table 2), show that out of the total, 13 patients (72.2%) passed away, 4 patients (22.2%) remained in a vegetative state, and only 1 patient (5.6%) (P16) experienced severe disability, regained consciousness, yet lost the capacity for autonomous movement.

## DISCUSSION

The randomized double-blind, crossover experiment found no effects of one session iTBS on the CRS-R scores of the patients with VS. There was a slight fluctuation of total CRS-R scores of five patients during the real stimulation and of three patients during the sham stimulation. It can be interpreted as a normal deviation of the CRS-R assessment, caused by fluctuation of patients’ behaviour or physical conditions. The fluctuation of CRS-R before and after sham stimulation also confirms this. Compared with one week’s diagnosis (at the beginning of experiment), only one patient (Patient 6) showed an arousal change (1 point in arousal sub-scale). However, it does not absolutely represent an improvement of consciousness, as the iTBS at the frontal region may have also evoked the muscle activities of the patient’s eyes. Thus, we suggest that the sign of spontaneous opening eyes may be induced by stimulation effects on the muscle.

The ‘uselessness’ of one session’s iTBS on CRS-R is consistent with findings in tDCS and rTMS studies. A single session of rTMS (10 Hz) did not induce any clinical improvement at the group level in patients with VS [30]. Our previous study also showed no changes of CRS-R after one session of tDCS over DLPFC in patients with VS [31]. However, although no consciousness improvement has been captured by CRS-R, we still should not conclude that iTBS is non-effective for treating patients with VS. Multi-sessions’ setups or more powerful protocols may provide a different picture.

In addition to a behavioural assessment, we applied the EEG measures to assess electrophysiological effects of iTBS on the brains of patients with VS. In comparison with the sham stimulation, the real iTBS significantly increased the complexity (increased of PE values) of the EEG activities. At a temporal dimension, the PE significantly increased after 30 minutes of the stimulation and then returned to the baseline level in one hour. In terms of space, the iTBS induced changes of complexity mainly located at left frontal electrodes, which surround the target site of iTBS. There was no significant change of PE value at any other electrodes, indicating a local modulation effects of iTBS on the neural nonlinear behaviours of the patients with VS. Coincidentally, in our previous study, the cortical excitability measures found that the effects of tDCS cannot be diffused from underneath the stimulated area to other brain regions for patients with VS [14]. Therefore, it could be suggested that iTBS or tDCS can significantly locally modulate the neural activities of patients with VS, but this could be invalid at the global scale. The ‘regional’ modulation effect may be the explanation behind why the more complex neural activities were not accompanied by and improvement of consciousness level for the patients with VS.

In addition to altering the complexity, iTBS enhanced the inter-hemisphere connectivity of the patients with VS. This enhancement was transient at the frontal region. It suggests that the effects of iTBS to reverse the integration of brain activities of the patients. Considering a serious impairment of functional inter-region connectivity in patients with VS, a reconstruction of neural networks may be helpful for their consciousness rehabilitation. But, the enhancement of functional connectivity induced by iTBS did not accompany the arising of consciousness levels. It is commonly inconsistent, because a matchup always exists between brain functional connectivity and the consciousness levels in patients with DOC [32–34]. We considered that the rising smaller and local functional connectivity arising induced by one session’s iTBS may finally fail to cause a transition of conscious state in such patients. However, although iTBS did not improve the consciousness of the patients, it still presents a better performance than the tDCS for modulating brain activities of patients with VS. In a previous study, we assessed the modulation effects of one session’s tDCS on the brain activities of patients with DOC [31]. It revealed non-effects of tDCS on the brains’ functional connectivity in patients with VS.

A 6-month GOS-E assessment revealed predominantly unfavorable long-term functional outcomes among patients with VS, with 17 patients (94.4%) experiencing poor outcomes (72.2% deceased during the trial, and 22.2% remaining in an implanted state at the trial’s conclusion), while only 1 patient (P16) exhibited a favorable outcome, aligning with findings from Faugeras’ study [35]. The good functional outcome in patient 16 does not necessarily translate to an improvement in functional status for vegetative state patients following one session of iTBS. This variation could be influenced by factors such as the patient’s underlying condition and the duration of their vegetative state. It is noteworthy that individuals experiencing consciousness disturbances post-traumatic brain injury tend to have a better outcome than those with different etiologies, with a shorter time to onset of the vegetative state correlating with a more positive outcome.

## CONCLUSIONS

Overall, this is the first study to examine the neuro-modulation effects of iTBS on patients with VS. Controlled by the sham stimulation, the effects of iTBS were assessed through behavioural assessments, EEG complexity and functional connectivity. The results showed non-effects of one session’s iTBS on the conscious behaviour of the patients with VS. But, an increase of PE around the target site was found within half an hour following iTBS. Inter-hemisphere connectivity was temporally and locally strengthened by iTBS. Although limited by the sample size, it still presents a decent performance on modulating the brain activities of patients with VS. Therefore, this study provides a pilot electrophysiological foundation that suggests that conducting multi-sessions of iTBS is valuable for exploring its treatment effects in patients with VS.
